# Validity of the international physical activity questionnaire short form (IPAQ-SF): A systematic review

**DOI:** 10.1186/1479-5868-8-115

**Published:** 2011-10-21

**Authors:** Paul H Lee, Duncan J Macfarlane, TH Lam, Sunita M Stewart

**Affiliations:** 1 FAMILY: A Jockey Club Initiative for a Harmonious Society, School of Public Health, Li Ka Shing Faculty of Medicine, University of Hong Kong, 21 Sassoon Road, Hong Kong; 2 Institute of Human Performance, University of Hong Kong, 111-113 Pokfulam Road, Hong Kong; 3 Department of Psychiatry, University of Texas Southwestern Medical Center at Dallas, 5323 Harry Hines Boulevard, Dallas, Texas 75390, USA

## Abstract

**Background:**

The International Physical Activity Questionnaire - Short Form (IPAQ-SF) has been recommended as a cost-effective method to assess physical activity. Several studies validating the IPAQ-SF have been conducted with differing results, but no systematic review of these studies has been reported.

**Methods:**

The keywords "IPAQ", "validation", and "validity" were searched in PubMed and Scopus. Studies published in English that validated the IPAQ-SF against an objective physical activity measuring device, doubly labeled water, or an objective fitness measure were included.

**Results:**

Twenty-three validation studies were included in this review. There was a great deal of variability in the methods used across studies, but the results were largely similar. Correlations between the total physical activity level measured by the IPAQ-SF and objective standards ranged from 0.09 to 0.39; none reached the minimal acceptable standard in the literature (0.50 for objective activity measuring devices, 0.40 for fitness measures). Correlations between sections of the IPAQ-SF for vigorous activity or moderate activity level/walking and an objective standard showed even greater variability (-0.18 to 0.76), yet several reached the minimal acceptable standard. Only six studies provided comparisons between physical activity levels derived from the IPAQ-SF and those obtained from objective criterion. In most studies the IPAQ-SF overestimated physical activity level by 36 to 173 percent; one study underestimated by 28 percent.

**Conclusions:**

The correlation between the IPAQ-SF and objective measures of activity or fitness in the large majority of studies was lower than the acceptable standard. Furthermore, the IPAQ-SF typically overestimated physical activity as measured by objective criterion by an average of 84 percent. Hence, the evidence to support the use of the IPAQ-SF as an indicator of relative or absolute physical activity is weak.

## Introduction

With changing social and economic patterns all over the world, sedentary lifestyles have become a worldwide phenomenon [1,2]. Sedentary lifestyles are associated with increased obesity, type 2 diabetes [3], and cardiovascular disease [4], and hence the promotion of active lifestyles is an important public health priority. To monitor trends and evaluate public health or individual interventions aiming at increasing levels of physical activity, reliable and valid measures of habitual physical activity are essential. Several routine instruments are available to measure physical activity, including self-report questionnaires, indirect calorimetry, direct observation, heart rate telemetry, and movement sensors [5]. All of these methods have well-known limitations [6], and for physical activity there is currently no perfect gold-standard criterion [7,8]. Movement sensors such as accelerometers have grown in popularity recently as a measure of physical activity [9], not only due to their objective measurements, but also due to their relatively small and unobtrusive size. Nevertheless, due to their high costs, accelerometers are not usually practical in large-scale cohort studies and instead questionnaires are frequently used to obtain physical activity data [10,11].

There are numerous available choices for questionnaires measuring physical activity [12]. Recent reviews have documented 85 self-administered physical activity questionnaires for adults [13], 61 for youth [14], and 13 for the elderly [15]. Many of these questionnaires have study-specific items and time referents, severely limiting the potential for comparisons across different studies. For example, the Synchronized Nutrition and Activity Program [16] measures activity relevant only to primary school children, and contains items that are not common across broad sectors of the population. The International Physical Activity Questionnaire (IPAQ) was developed to address these concerns by a group of experts in 1998 to facilitate surveillance of physical activity based on a global standard [17]. The IPAQ has since become the most widely used physical activity questionnaire [13], with two versions available: the 31 item long form (IPAQ-LF) and the 9 item short form (IPAQ-SF). The short form records the activity of four intensity levels: 1) vigorous-intensity activity such as aerobics, 2) moderate-intensity activity such as leisure cycling, 3) walking, and 4) sitting. The original authors recommended the "last 7 day recall" version of the IPAQ-SF for physical activity surveillance studies [17], in part because the burden on participants to report their activity is small.

A common analysis method used to demonstrate questionnaire validity is to correlate self-reported activity data from the IPAQ-SF with data from an objective measurement device(s), both of which are obtained over exactly the same time period (concurrent validity). Another common method is to compute the absolute differences between the objective and self-reported measure. Both methods are essential in determining the validity of the IPAQ-SF, and a systematic review of the analyses that have been used to validate the IPAQ-SF would therefore be useful in assessing the merits of using the IPAQ-SF in epidemiological studies.

The first comprehensive validation of the IPAQ-SF was conducted across 12 countries, and reported correlations (all correlations reported were Spearman ρ's for the last 7 day's report) with the uniaxial CSA model-7164 accelerometer. A wide range of Spearman correlations, ρ = 0.02 (Sweden) - 0.47 (Finland), raised concerns of variability in validity in different populations. Variability in reported validity may be caused by several factors such as the demographic and cultural backgrounds of the participants, the way the information requested is processed and delivered, as well as variations in the "criterion gold-standard" used for objective comparison. Criterion measures used for IPAQ-SF validation have included the actometer [18], accelerometer [19] and pedometer [20], yet only one study has used the expensive doubly labeled water technique [21] as a criterion even though it has been recommended and is considered the most accurate objective measurement of physical activity [8,22]. In addition to traditional measures of physical activity, various fitness measures (e.g. maximum oxygen uptake, VO2max [23]) have also been used as a reference standard to compare the IPAQ-SF because physical activity is strongly associated with cardiorespiratory fitness [24]. Several of the objective measures yield different indices of activity, and the findings regarding validity may vary according to which index and objective measure is used as the standard, for example, both time spent in physical activity and raw count data have been used as a measure of physical activity from accelerometer [25]. Variations also occur in how the objective measured data were transformed, for example the transformation algorithm from raw accelerometer data to time spent in moderate to vigorous physical activity [26,27]. There have also been inconsistencies in the reporting of "total physical activity" from IPAQ-SF data, with studies using units involving metabolic equivalent task (MET), time spent in activity, or simply a trichotomized variable indicating the adequacy of physical activity [28]. The IPAQ-SF instrument may also be better at capturing activity of some intensity level but not others, e.g., vigorous rather than moderate activity. Because the variability shown in the IPAQ-SF validity from these international studies has not been collated and systematically examined, we reviewed the effect of these sources on IPAQ-SF validity.

The IPAQ was first published with its validation based on a 12-country sample, and the authors recommended using the short form which measured physical activity by self-report over the previous 7 days [17]. Since that time, more validation studies have been published for this short-form than for any other physical activity questionnaires [13]. Despite the popularity of the IPAQ-SF and its widely accepted high reliability [13,17], there has been no systematic review of its validity. Van Poppel et al. [13] have published a review of physical activity questionnaires used in adults, but included only four studies of the IPAQ-SF. Hence, a more comprehensive review of the IPAQ-SF is needed using data from the English language literature, with a focus on the variability of its relationship with the various validation measures as well as its absolute accuracy.

This paper has two objectives: (1) to review the analyses used in the IPAQ-SF validation studies, and (2) to consider possible explanations for differences between studies. For the first objective, we reviewed the studies validating the IPAQ-SF as a relative measure (i.e. studies that show a correlation with objective measures of physical activity) and/or an absolute measure (i.e. studies that compare levels of physical activity obtained by the IPAQ-SF against levels from an objective measure) of physical activity level. For the second objective, we examined whether the demographics of different samples, the indices derived from objective standards or the IPAQ-SF, or additional moderators which had contributied to the different levels of validity reported. Since the IPAQ-SF has been consistently shown to have a high reliability (ranging from 0.66 to 0.88) [17,20,25], we will not study this property here. We examined studies that sought to validate both (a) the overall physical activity score from the IPAQ-SF, as well as (b) those that focused on restricted information from the scale, e.g., different levels of intensity (vigorous activity, moderate activity and walking).

## Methods

### Literature search

We searched in PubMed and Scopus for papers examining the validity of the IPAQ-SF through November 2010, using the keywords "IPAQ AND (validity OR validation)". Additional papers were gathered by searching the reference lists from the searched papers.

### Inclusion criteria

Each paper had to satisfy the following criteria in order to be included in our review. First, the validation had to be of the short form against an objective physical activity measuring device, (e.g., accelerometer or pedometer), or an objective fitness/anthropometric measure (e.g. VO2max or % body fat). Validation papers of the IPAQ-SF against self-reported measures such as other physical activity questionnaires or log-books, and reliability studies without validity information were not included. Second, the article was published in English.

### Search result

The search in PubMed and Scopus yielded 51 and 56 papers respectively (with a total of 59 unique papers). Of these, 38 papers were excluded for the following reasons: 13 papers used the IPAQ long form; 11 papers validated other measures using the IPAQ-SF as the standard; five papers were not in English; three papers validated a modified version of the IPAQ-SF; three papers were applications of the IPAQ-SF; one paper reviewed properties of physical activity questionnaires among the elderly; one was a comment article and one was a qualitative study translating the IPAQ-SF. Two more papers were identified through the reference lists of the papers reviewed [28,29]. Overall, 23 studies were reviewed in the present paper [17-20,23,25,28-44] and their general characteristics are presented in Table 1.

**Table 1:** General characteristics of 23 included studies

Reference	Place of study	Targeted population (general population if not specified)	N	% Male	Mean age
Scheeres 2009 [18]	The Netherlands	Chronic fatigue syndrome	226	26.1%	37.0
Kaleth 2010 [33]	USA	Fibromyalgia patients	30	10.0%	49.1
Lachat 2008 [35]	Vietnam	Grade-11 students	227	NA	16.0
Mader 2006 [36]	Switzerland	German-speaking	35	62.9%	54.7
Dinger 2006 [25]	USA	College students	123	26.0%	20.8
Ekelund 2006 [31]	Sweden		185	47.0%	41.8
Vandelanotte 2005 [29]	The Netherlands		53	NA	NA
Craig 2003 [17]	12 countries		716	49.2%	37.3
Wolin 2008 [39]	USA	African-Americans	142	35.9%	44.0
Rangul 2008 [23]	Norway	Secondary school students	67	44.8%	14.9
Kurtze 2008 [39]	Norway	Men, age 20-39	108	100%	32.4
Macfarlane 2007 [19]	Hong Kong, China		49	61.2%	28.7
Faulkner 2006 [32]	Canada	Schizophrenia patients	35	63.0%	39.7
De Cocker 2009 [30]	Belgium		288	48.3%	38.7
Deng 2008 [20]	Guangzhou, China		224	33.9%	65.2
Cust 2009 [40]	Australia		177	NA	NA
Timperio 2004 [42]	Australia		285	NA	NA
Kolbe-Alexander 2006 [43]	South Africa		42	41.0%	66.8
Papathanasiou 2010 [37]	Greece		218	51.8%	23.0
Ramirez-Marrero 2010 [38]	Puerto Rico	Hispanic patients with HIV	58	60.3%	46.5
Ishikawa-Takata 2008 [28]	Japan		150	49.3%	38.7
Egeland 2008 [44]	Canada	Cree Territory	161	59.0%	38.4
Fogelholm 2006 [41]	Finland	Finnish Defence Forces	967	100%	29.0

### Data extraction

The following information was extracted from papers included in the review: (1) validity data, i.e. a) the correlation between different levels of intensity of the IPAQ-SF (vigorous activity, moderate activity, walking) and their corresponding time spent measured by the objective standard; and b) whether raw values were reported and if so, the percentage difference between the IPAQ-SF and the objective standard (with the objective standard used as the reference). (2) In addition, the following potential sources of variability in findings were noted: a) the country of study, the target population (if specified), and the size and demographics of the sample; b) the objective physical activity measure(s) and/or the fitness measure(s) used as the objective standard; c) the unit of measurement of the objective standard (for example, raw accelerometer counts, metabolic equivalent task (MET), total time spent on physical activity, MET-transformed energy expenditure, etc.), and the cutoff levels used to categorize activity into moderate and vigorous activity; d) the correlation between the IPAQ-SF total activity level (MET, time spent, or any novel definition introduced by the investigators) and the objective standard; and e) potential factors influencing the relationships reported between the IPAQ-SF and the objective physical activity or fitness measures.

### Data synthesis and analysis

Results of the 23 studies were synthesized into four categories: (1) validity of the IPAQ-SF to measure overall physical activity; (2) validity of the IPAQ-SF to measure specific levels of physical activity; (3) accuracy of IPAQ-SF; and (4): factors that might relate to the variability of IPAQ-SF validity.

Table 2 presents information from 16 studies [17-20,23,25,29-37,39] regarding the standard, unit, and activity value used, and the correlation of the objective standard with the IPAQ-SF and its associated effect size in the different studies examining physical activity on a continuum. Table 3 presents the remaining 7 studies which did not present information from continuous measures of physical activity [28,41], did not present information for the whole sample but in subgroups [40,43], and presented only correlations for specific intensity [38,42,44]. Most studies examined the validity of the IPAQ-SF by reporting the Spearman ρ for the relationship between the scale and the objective physical activity measure(s) and/or the fitness measure(s). Using Ferguson's [45] guideline for effect size interpretation for the ρ, values of 0.2, 0.5, and 0.8 were described as small, moderate, and large effects respectively. Effect sizes below 0.2 are reported in this paper as negligible. Using Terwee and colleagues' guidelines [8], effect sizes above 0.5 were considered acceptable for correlations against objective activity measuring devices, and above 0.4 for fitness measures. Table 3 presents the studies that examined the validity of the IPAQ-SF by examining the correlation between the scale and the physical activity/fitness measures at different levels of intensity. This table includes information from 15 studies [20,23,25,28,30,34-38,40-44], 8 of which [20,23,25,30,34-37] presented overlapping data from continuous measures of physical activity are also included in Table 2. For studies that examined the validity of IPAQ-SF at specific levels of intensity, the correlation between the IPAQ-SF and the objective physical activity measures are shown in Table 3. Table 4 presents under- and over-reporting of physical activity by the IPAQ-SF compared to objective data from the accelerometer. Six studies provided information relevant to this aim.

**Table 2:** Performance of the overall IPAQ-SF: Correlations between the objective measures and the IPAQ-SF overall physical activity levels (MET score, time spent, or novel definition by investigators) from 16 studies

Reference	Objective standard	Objective standard unit	IPAQ-SF total activity value used (Total PA min/wk: 2 × time spent on vigorous + moderate + walking, MET min/wk: 8 × vigorous + 4 × moderate + 3.3 × walking)	ρ	Effect size (< 0.2: negligible; 0.2 to 0.49: small; 0.5 to 0.79: moderate; ≥0.8: large)
Scheeres 2009 [18]	Actometer	Actometer score	MET min/wk	0.33	Small
Craig 2003 [17]	Accelerometer	Count	MET min/wk	0.30	Small
Dinger 2006 [25]^1^	Accelerometer	Count	Total PA min/wk	0.21	Small
Vandelanotte 2005 [29]	Accelerometer	Count	Total PA min/wk	0.38	Small
Ekelund 2006 [31]^2^	Accelerometer	Count	MET min/wk	0.34	Small
Kaleth 2010 [33]^3^	Accelerometer	Count	Total PA min/wk	0.33	Small
Lachat 2008 [35]	Accelerometer	Count	MET min/wk	0.21	Small
Mader 2006 [36]^4^	Accelerometer	Count	MET min/wk	0.39	Small
Wolin 2008 [39]	Accelerometer	Total PA min/wk#	MET min/wk	0.26	Small
Rangul 2008 [23]^5^	Accelerometer	TEE	3 categories‡	0.09	Negligible
Kurtze 2008 [34]^6^	Accelerometer	AEE	MET min/wk	0.26	Small
Macfarlane 2007 [19]	Accelerometer	MET min/wk (Freedson)	MET min/wk	0.09	Negligible
Dinger 2006 [25]^1^	Accelerometer	MET min/wk (Freedson)	Total PA min/wk	0.23	Small
Ekelund 2006 [31]^2^	Accelerometer	MET min/wk (Freedson)	MET min/wk	0.30	Small
Faulkner 2006 [32]	Accelerometer	Total PA min/wk	Total PA min/wk	0.37	Small
Deng 2008 [20]	Pedometer	Count	MET min/wk	0.33	Small
Dinger 2006 [25]^1^	Pedometer	Count	Total PA min/wk	0.25	Small
De Cocker 2009 [30]	Pedometer	Count	Total PA min/wk	0.28	Small

Reference	Fitness measure	Objective standard unit	IPAQ-SF total activity value used	ρ	Effect size

Papathanasiou 2010 [37]	Treadmill	Maximum time endured	Total PA min/wk	0.36	Small
Kaleth 2010 [33]^3^	6-min walk test	Walking distance	Total PA min/wk	0.16	Negligible
Rangul 2008 [23]^5^	VO_2max_	ml/kg/min	3 categories‡	0.32	Small
Kurtze 2008 [34]^6^	VO_2max_	ml/kg/min	MET min/wk	0.30	Small
Mader 2006 [36]^4^	VO_2max_	ml/kg/min	MET min/wk	0.24	Small

AEE: average energy expenditure

TEE: total energy expenditure

MET: metabolic equivalent task

MET min/wk (Freedson): moderate PA: 1952≤ count/min ≤5724, vigorous PA: count/min > 5724

Studies cited more than once have been identified with the same superscript number

3 categories‡: novel definition [23] of: low, moderate, high

#: Accelerometer counts were transformed to AEE, and then AEE was transformed to time spent on moderate and vigorous activity

**Table 3:** Performance of the IPAQ-SF within specific levels of activity: Correlations between objective/fitness measures and physical activity sub-scores at different levels of intensity from 15 studies

Reference	Objective standard	Objective standard unit	IPAQ-SF intensity (min/wk) (ρ)			Re-categorization by the investigators
			Vigorous	Moderate	Walking	
Ishikawa-Takata 2008 [28]	Doubly labeled water	TEE				/(3 categories)‡
		PAL				* (3 categories) ‡

Lachat 2008 [35]	accelerometer	Count	0.29	-0.01		
Mader 2006 [36]^1^	accelerometer	Count	-0.18	0.23	0.42^b^	0.43 (moderate + walking)
Dinger 2006 [25]^2^	accelerometer	Step	0.30	0.14		
Kolbe-Alexander 2006 [43]^3^	accelerometer	Count		0.37†	0.57†^a^	
				0.08††	0.42††^b^	
Rangul 2008 [23]^4^	accelerometer	TEE				0.09 (3 categories) ‡
		PAL				0.03 (3 categories) ‡
Kurtze 2008 [34]^5^	accelerometer	AEE	0.05	0.16		
		PAL	0.08	0.14		
Mader 2006 [36]^1^	accelerometer	Vigorous PA min/wk (Swartz)	-0.03			
Dinger 2006 [25]^2^	accelerometer	Vigorous + moderate 10-min bout	0.44^b^	0.19		
		Vigorous PA min/wk	0.47^b^			
Cust 2009 [40]^6^	accelerometer	Vigorous PA min/wk	0.28#			
			0.32##			
Timperio 2004 [42]^7^	accelerometer	Vigorous PA min/wk	0.15¶			
			0.28¶¶			
Kolbe-Alexander 2006 [43]^3^	accelerometer	Vigorous count (Freedson)	0.43†^b^			
			0.05††			

Ramirez-Marrero 2010 [38]^8^	accelerometer	Moderate PA min/wk (Freedson)	0.23	-0.03		
		Vigorous + moderate min/wk (Freedson)				0.15 (Vigorous + moderate min/wk)
Mader 2006 [36]^1^	accelerometer	Moderate PA min/wk (Swartz)		0.38	0.27	0.39 (moderate + walking)
Dinger 2006 [25]^2^	accelerometer	Moderate PA min/wk		0.23		
Cust 2009 [40]^6^	accelerometer	Moderate PA min/wk		0.34#		0.32# (moderate + walking)
				0.01##		0.08## (moderate + walking)
Timperio 2004 [42]^7^	accelerometer	Moderate PA min/wk		0.13¶		
				0.27¶¶		
Kolbe-Alexander 2006 [43]^3^	accelerometer	Moderate PA min/wk		0.31†	0.56†^a^	
				-0.09††	0.08††	
Ramirez-Marrero 2010 [38]^8^	pedometer	Count	0.16	0.76^a^		0.18 (vigorous + moderate min/wk)
De Cocker 2009 [30]	pedometer	Count	0.20	0.33	0.15	
Deng 2008 [20]	pedometer	Count	-0.09	0.05	0.51^a^	
Dinger 2006 [25]^2^	pedometer	Count	0.38	0.17		

Reference	Fitness measure	Fitness measure unit	IPAQ-SF intensity (min/wk) (ρ)			Re-categorization by the investigators
			Vigorous	Moderate	Walking	

Papathanasiou 2010 [37]	Treadmill	Maximum time endured	0.43^b^	0.16		
Rangul 2008 [25]^4^	VO_2max_	Walking distance				0.32 (3 categories)
Kurtze 2008 [34]^5^	VO_2max_	ml/kg/min	0.41^b^	0.19		
Mader 2006 [36]^1^	VO_2max_	ml/kg/min			0.29	
Fogelholm 2006 [41]	VO_2max_	ml/kg/min	*‡‡‡			* (5 categories) ‡‡
Egeland 2008 [44]	Body fat	Percentage	-0.26			
Fogelholm 2006 [41]	Sit-ups	Maximum number	*‡‡‡			* (5 categories) ‡‡
	Push-up	Maximum number	*‡‡‡			* (5 categories) ‡‡
	Squats	Maximum number	*‡‡‡			* (5 categories) ‡‡

AEE: average energy expenditure

TEE: total energy expenditure;

PAL: physical activity level (TEE/basal metabolic rate)

MET: metabolic equivalent task

MET min/wk (Swartz): moderate PA: 574≤ count/min ≤4945, vigorous PA: count/min > 4945

MET min/wk (Freedson): moderate PA: 1952≤ count/min ≤5724, vigorous PA: count/min > 5724

Studies cited more than once have been identified with the same superscript number

3 categories‡: novel definition [23] of: low, moderate, high

5 categories‡‡: novel definition [36] of five quintiles according to IPAQ-SF total MET score (‡‡)/time spent on vigorous activity (‡‡‡)

^a^: moderate effect size (0.5 - 0.79)

^b^: approaching moderate effect size (0.4 - 0.49)

†/††: male/female

#/##: high/low confidence

¶/¶¶: with/without logbook

*: significant (*p <*0.05) between-category difference from ANOVA test

/: nonsignificant (*p >*0.05) between-category difference from ANOVA test

**Table 4:** Discrepancy between concurrent IPAQ-SF and accelerometer data computed using results from 6 studies

Reference	Cutoff used	IPAQ-SF MET-min/wk	Accelerometer MET-min/wk	Over-report % (based on accelerometer as criterion)
Lachat 2008 [35]	Trost	1512	812	86%
Macfarlane 2007 [19]	Freedson	3931	1440	173%
Dinger 2006 [25]	Freedson	2607	1299	101%
Mader 2006 [36]	Swartz	6929	5088	36%
Timperio 2004 [42]	Freedson	2987	1275	134%

Ekelund 2006 [31]	Freedson	1032	1430	-28%

MET: metabolic equivalent task

Trost: MET = 2.757+(0.0015 × counts/min) -0.08957 × age)-(0.000038 × counts/min×age)

Swartz: moderate PA: 574≤ count/min ≤4945, vigorous PA: count/min > 4945

Freedson: moderate PA: 1952≤ count/min ≤5724, vigorous PA: count/min > 5724

## Results

### Validity of the overall IPAQ-SF: overall physical activity level

These data are presented in Table 2. The IPAQ-SF showed negligible to small correlations in total activity level with objective measuring devices (range of ρ = 0.09 [19] to 0.39 [36], median = 0.29). Among the 18 correlations reported for objective measuring devices [17 - 20, 23, three reported in 25, 29, 30, two reported in 31, 32 - 35, 39], 16 of them were regarded as small and the others were negligible. In general, the correlation of the IPAQ-SF with accelerometer data (range of ρ = 0.09 [19] to 0.39 [36], median = 0.28) was the same with that of the pedometer (range of ρ = 0.25 [25] to 0.33 [20], median = 0.28) and actometer (ρ = 0.33 [18]).

With fitness measures (VO2max, maximum treadmill time, and 6-minute walk test reported in the lower section of Table 2), the correlations with the IPAQ-SF total activity level were small in four of the five studies (range of ρ = 0.16 [33] to 0.36 [37], median = 0.30). Only one study validated the IPAQ-SF against anthropometric measures, which reported a small correlation between the IPAQ-SF and body fat percentage (ρ = -0.19 [44], not shown in any tables).

In the only study using doubly labeled water as the criterion measure [28], the validity of the IPAQ-SF was assessed by categorizing participants into insufficiently active, sufficiently active, and highly active based on their IPAQ-SF scores (Table 3). The total energy expenditure (TEE) and physical activity level (PAL) (both measured using doubly labeled water) were then compared across the three categories. TEE and PAL in the highly active participants were significantly higher than that of the other two groups, and the authors concluded that highly active participants could be correctly identified, and distinguished from inactive participants using the IPAQ-SF, but other discrimination was poor [28].

### Validity of the IPAQ-SF: specific levels of intensity

These data are presented in Table 3. Three studies [20,38,43] reported moderate to large correlations (ρ ≥0.5) for one of the different levels of intensity (vigorous activity, moderate activity, and walking) (superscript a in column 4-6 of Table 3). Of the four correlations [20, 38, two reported in 43] in the moderate range or higher (ρ ≥ 0.5), three [20, two reported in 43] were correlations related to walking time and the remaining one [38] related to moderate activity. All the above four correlated IPAQ-SF against accelerometer or pedometer values [20, 38, two reported in 43]. In addition, two studies [36,43] reported values in the 0.40 to 0.49 range for time spent on walking and accelerometer count. Time spent on walking seemed to correlate best with accelerometer/pedometer counts.

Of the five remaining studies [25,34,36,37,43] (superscript b in column 4-6 of Table 3) reporting correlations approaching the moderate level (ρ = 0.40 - 0.49), all measured activity at the vigorous level; two were correlations between vigorous activity time and fitness measures (VO2max [34] and maximum treadmill time [37]), and the other three were for vigorous time spent measured against accelerometer data [25,36,43]. As the correlation for validation against fitness measures is recommended as ρ = 0.40, there was some support for the validity of the IPAQ-SF in measuring vigorous activity. However, it should be noted that these represent only a third of the correlations reported against the fitness measures.

### Accuracy of the IPAQ-SF

Table 4 shows the accuracy of the IPAQ-SF. Six studies provided the amount in physical activity measured by the IPAQ-SF and objective data [19,25,31,35,36,42], but surprisingly, none of them computed the percentage of over- or under-reporting of physical activity, or used the absolute difference as an indicator of validity. Furthermore, standard deviations were not provided by these studies, making it impossible to compute the effect size for the differences between the IPAQ-SF and the objective device. Under-reporting of physical activity (-28%) was present in only one study [31], but in the other five studies [19,25,35,36,42], over-reporting by the IPAQ-SF of 106 percent on average when compared to the accelerometer was found (range 36 - 173%).

### Factors that might relate to variability of validity findings

#### Demographics

None of the demographic characteristics, including place of study, targeted population, sample size, male-female ratio, and age, seemed to be related to differences in validity between the IPAQ-SF and the criterion measure (Tables 1 and 2).

#### Objective standard used for validation

Fifteen studies used an objective device that monitored body motion [17-20,25,29-32,35,38-40,42,43], two examined scores against a physical fitness measure [37,41], four used both an objective device and a physical fitness measure [23,33,34,36] and one compared findings against anthropometric measures [44] (Tables 2 and 3). Of those reporting data from motion-sensing devices, one of them used the actometer, two used a pedometer, and fifteen used an accelerometer. Two of them used both a pedometer and an accelerometer. Notably, only one study used doubly labeled water [28] (Table 3), the recommended criterion for validation [8,22] to assess the validity of the IPAQ-SF.

#### Indices from objective standards used for validation

The third columns of Tables 2 and 3 indicate the unit used in the analyses. For the accelerometer device (excluding pedometers), and for the fitness measures, several different units were used and were not consistent across studies. Of the seventeen studies using an accelerometer as the objective standard (8 in Table 2[18-20,29,31-33,39], 4 in Table 3[38,40,42,43], and 5 in both [23,25,34-36]), four types of units were commonly reported (with some studies reporting multiple different units). These included (i) raw accelerometry counts without transformation (Counts [17,25,29,31,33,35,36,40,43]), (ii) count data to energy expenditure (TEE/AEE/PAL [23,34,39]), (iii) MET scores (MET min/wk [19,25,31,32,36,38,40,42]), and (iv) time spent (Total PA min/wk [25,31,36,38-40,42,43]). In addition to the variability of units used for reporting accelerometer data, there was also a great variability in the cutoffs used to transform the accelerometer data into MET min/wk. Three different cutoffs (Freedson [26], Swartz [27], and Trost [46]) were used among the aforementioned validation studies, yet overall, no pattern of difference in correlations was evident based on the use of the different cutoffs.

Nevertheless, this was not the case for the absolute discrepancy between the IPAQ-SF and the accelerometer scores (reported in Table 4). The only study using the Swartz cutoffs ([27], moderate PA: 574≤ count/min≤4945, vigorous PA: count/min > 4945) yielded an over-report of 36%, which appears relatively small compared with the average of 95% for the four studies [19,25,31,42] using the Freedson cutoffs (moderate PA: 1952≤ count/min≤5724, vigorous PA: count/min > 5724) (Table 4). In theory, the Swartz cutoffs will yield a lower MET score than the Freedson cutoffs, because some of the time spent on moderate activity classified by the Swartz cutoffs (574≤ count/min < 1952) may be classified as inactive by the Freedson cutoffs, so that total time spent computed using the Swartz cutoffs will be higher than that using the Freedson cutoffs. Note that it is impossible to conclude that the Swartz's cutoffs are more appropriate simply because they reduce the over-report of the IPAQ-SF, as the true level of physical activity is not known. As the Trost's cutoffs depend on the age of the participants, no direct comparison to the other two cutoffs can be made. It is of interest that no published study has yet compared IPAQ-SF with the more recent weighted-accelerometer cutoffs suggested by Metzger et al [47].

#### Indices from the IPAQ-SF

Values obtained from the IPAQ-SF have also been used in different ways in the various studies. Of the sixteen studies that computed the total physical activity from the IPAQ-SF (Table 2), six [25,29,30,32,33,37] used total time spent (Total PA min/wk), nine [17-20,31,34-36,39] transformed the total time spent to MET scores (MET min/wk), and one [23] used a novel trichotomized variable indicating the adequacy of physical activity (3 categories). Again, no pattern across the correlations was evident based on the use of these different indices.

#### Other potential moderators

Two studies aimed at finding potential factors influencing the validity of the IPAQ-SF. One group studied the relationship between the participant's confidence in accurately recalling physical activity on the IPAQ-SF [40], whilst the second group examined whether keeping physical activity logbooks improved the validity of the IPAQ-SF report [42]. The resultant correlations ranged from 0.15 to 0.30, whilst the confidence ratings and the act of completing daily logbooks did not influence the relationship between the IPAQ-SF and the objective measures. Although logbooks did not improve IPAQ-SF validity, one IPAQ-SF validation paper written in Chinese [48] showed that using a logbook to impute missing accelerometer data could yield an acceptable IPAQ-SF validity (Pearson correlation = 0.63, not shown in tables).

## Discussion

A recently published checklist of attributes of physical activity questionnaires [8] suggested that correlations of 0.5 for moderate and vigorous activity and 0.4 for total energy expenditure or fitness should be the standard for an acceptable self-reported physical activity questionnaire. Despite the very broad range of methods reported in Table 2, the findings were quite consistent: the correlation between the IPAQ-SF overall scale and any index never reached the standard of 0.50 [13]. When the self-reported data from the IPAQ-SF was restricted to a narrower ranges of activity levels (Table 3), there were nominally more promising results. The total time spent derived from the IPAQ-SF for walking showed small-to-moderate correlations with step counts obtained from objective devices, with about one third of the correlations falling into the acceptable range. This was not the case for moderate or vigorous activity, which correlated weakly with measures from objective devices, yet time spent on vigorous activity correlated moderately well with fitness measures, with most of these correlations reaching an acceptable level. In summary, only four (with superscript a) of 74 correlations reported (Tables 2 and 3) were in the recommended range of > 0.50 for a correlation with an objective device, and two (with superscript b) of 12 correlations reported (Tables 2 and 3) were in the recommended range of > 0.40 for a correlation with a fitness measure.

For walking activity, most studies validated the results against the accelerometer, although one correlated moderate activity against the pedometer, as moderate walking is often associated with a MET = 3.3 [49], which is considered by some to be within the moderate intensity range of 3-5.9 METs [26]. When examining absolute accuracy, few studies reported absolute scores, and none reported standard deviations so the effect size of the difference in findings between the objective measure and the IPAQ-SF could not be computed. The smallest discrepancy reported was an under-estimate by the IPAQ-SF of 28 percent, yet most of these studies reported an over-estimate by the IPAQ-SF and showed considerable variability and the overall mean over-estimate in these studies was 106 percent. Over-reporting of physical activity by the IPAQ-SF is not uncommon [50], and it remains a key limitation of most self-reported measures of physical activity [51].

### Future research directions

Only one study has validated the IPAQ-SF against doubly labeled water and despite the high cost, this criterion remains the recommended standard for studies comparing energy expenditure. Very few studies have evaluated the accuracy of the IPAQ-SF, i.e. the concordance of absolute values between the measure obtained by an objective physical device and that by the IPAQ-SF. It is recommended that further validation studies are needed using both research techniques.

The literature shows much variability in the reported units of activity used to compare against the IPAQ-SF data. For example, raw counts, MET scores, and time spent were used by researchers to report total activity levels derived from the accelerometer, with no consistency or apparent agreement. Greater consistency in the reporting of the accelerometry data would enhance future comparative studies. Furthermore, a variety of accelerometer cut-offs were used by different researchers to define categories of activity which alone would generate varying and incomparable results [52,53]. These accelerometer cut-offs were determined by calibrating accelerometer counts during specific activities (e.g. housework, recreation), and all were typically calibrated in samples from the United States [26,27,46]. If the cutoffs are to be truly adopted globally with accelerometry research, similar and standardized studies are needed from different cultures.

## Conclusions

Although the IPAQ-SF is recommended and widely used, our systematic review has found that in the large majority of validation studies only a small correlation with objective measures of activity was achieved. Nevertheless, there are a few exceptions, with vigorous activity and walking showing some acceptable correlations. Furthermore, the IPAQ-SF tends to overestimate the amount of physical activity reported compared to an objective device. As a result, the current evidence is fairly weak to support the use of the IPAQ-SF as either a relative, or as an accurate and absolute measure of physical activity, although its proven reliability shows it can be used with care in repeated measures studies, although the true magnitude of the change over time, if any, may not be accurate. Comparability of studies that wish to assess the validity of self-report questionnaires is achieveable if researchers use more consistent units and standardized categorization of intensity levels from accelerometry studies. Also, providing a distinction between validation strategies for relative and absolute interpretations of physical activity questionnaires is important.

## Competing interests

The authors declare that they have no competing interests.

## Authors' contributions

All authors read and approved the final manuscript. PHL conducted the literature review and the abstraction of study data, and drafted the manuscript. DJM and THL contributed to the study conceptualization and manuscript preparation, and provided critical editorial input to the interpretation of the data. SMS conceived of the study, contributed to the literature review, and contributed to the writing of the manuscript.
